# A20 Mutation Is Not a Prognostic Marker for Activated B-Cell-Like Diffuse Large B-Cell Lymphoma

**DOI:** 10.1371/journal.pone.0145037

**Published:** 2015-12-30

**Authors:** Hong Cen, Xiaohong Tan, Baoping Guo

**Affiliations:** Department of Chemotherapy, Tumor Hospital of Guangxi Medical University, Nanning, Guangxi, People’s Republic of China; National Institute of Genomic Medicine, MEXICO

## Abstract

**Background:**

Constitutive activation of nuclear factor κB (NF-κB) is a hallmark of activated B-cell-like diffuse large B-cell lymphoma (ABC-DLBCL). Mutations in the A20 gene activate NF-κB, but the prognostic value of A20 mutations in ABC-DLBLC is unclear.

**Purpose:**

To investigate the prognostic value of A20 mutation in ABC-DLBCL patients.

**Methods:**

The somatic mutation of A20 was investigated in 68 *de novo* ABC-DLBCLs by PCR/sequencing. The Kaplan-Meier method was used to estimate median overall survival (OS) and progression-free survival (PFS).

**Results:**

The A20 mutation rate in ABC-DLBCL patients was 29.4%. Complete remission rates were 35% and 45.8% in patients with and without A20 mutations, respectively (P = 0.410). In patients with and without A20 mutations, the median OS was 24.0 and 30.6 months, respectively (P = 0.58), and the median PFS was 15 and 17.4 months, respectively (P = 0.52). None of the differences between the patient groups were significant.

**Conclusions:**

Our findings suggested that the A20 mutation is a frequent event in ABC-DLBCLs. However, there was no significant difference in PFS and OS in patients with or without A20 mutations. Further study is required to completely exclude A20 somatic mutation as a prognostic marker in the ABC subtype of DLBLC.

## Introduction

Activated B-cell-like diffuse large B cell lymphoma (ABC-DLBCL) cells require constitutive NF-κB signaling for survival, whereas germinal center B (GCB) cells do not require constitutive NF-κB signaling for survival [1]. Interruption of NF-κB signaling selectively induces apoptosis of ABC-DLBCL cells [1, 2]. Recent studies have shown that B-cell receptors (BCRs) activate NF-κB and that chronic active BCR signaling is also required for survival of ABC-DLBCL cells [3]. A20, also known as tumor necrosis α (TNFα)-induced protein 3 (TNFAIP3), inhibits NF-κB activation by TNFα and Toll-like receptors [4–6]. Recently, somatic mutation of A20 gene which constitutively activate NF-κB had been reported by several investigators [7–9]. The mutation rate of A20 gene in ABC-DLBCL is relatively high and A20 somatic mutation was significantly associated with the ABC subtype of DLBCL [8, 9]. This study focus on whether there is a prognostic difference between ABC-DLBCL with or without A20 somatic mutation. This information could potentially further stratify patients in clinical treatment.

## Materials and Methods

### Patients

This study consisted of 68 de novo ABC-DLBCL patients treated at the Tumor Hospital of Guangxi Medical University between January 2010 and March 2012. The clinical characteristics of the patients are summarized in Table 1. The study was approved by the Ethics Committee of the Tumor Hospital of Guangxi Medical University. All participants signed written informed consent. All data were anonymized and de-identified.

**Table 1:** Clinical characteristics of patients with activated B-cell-like diffuse large B-cell lymphoma according to the presence of the A20 mutation.

Variable	Mutation status (n)		P value
	yes	no	
**Sex**			0.595
Male	11	23	
Female	9	25	
**Age (years)**			0.727
<60	15	34	
≥60	5	14	
**Stage**			0.659
I–II	8	22	
III–IV	12	26	
**LDH**			0.528
Normal	10	20	
Elevated	10	28	
**Number of extranodal sites**			0.667
<2	14	31	
≥2	6	17	
**PS status**			0.487
0–1	18	39	
2–4	2	9	
**BCL2**			0.532
+	17	37	
-	3	11	
**IPI score**			0.974
0–2	13	31	
3–5	7	17	
**Chemotherapy regimen**			1.000
CHOP	16	39	
R-CHOP	4	9	
**Complete remission**			0.410
Yes	7	22	
No	13	26	

LDH: lactate dehydrogenase; PS: performance status; IPI: international prognostic index; CHOP: cyclophosphamide, doxorubicin, vincristine, and prednisolone; R-CHOP: rituximab plus CHOP

### Tumor tissue

Formalin-fixed paraffin-embedded (FFPE) tissue specimens were obtained from the Department of Pathology at the Tumor Hospital of Guangxi Medical University. All specimens contained >50% tumor cells and adequate material for DNA extraction.

### Immunohistochemistry

All cases were diagnosed and classified in accordance with the 2008 criteria of the World Health Organization for tumors of hematopoietic and lymphoid tissue. Immunohistochemistry was routinely performed on FFPE tissue sections. The germinal center and ABC-DLBCL subgroups were diagnosed via immunohistochemical analysis of CD10, BCL6, and melanoma-associated antigen (mutated) 1 using the algorithm of Hans et al [10]. Cases were considered positive if 30% or more DLBCL cells expressed these markers.

### Mutation analysis

Genomic DNA was extracted from paraffin-embedded material using a QIAamp DNA mini kit (QIAGEN, Valencia, CA). The complete coding sequence of the A20 gene was analyzed via polymerase chain reaction (PCR) amplification and direct sequencing of genomic DNA. The primers used for amplification and sequencing of each exon are shown in S1 Table. A20 mutations were confirmed by sequencing of both strands in independent PCR products. Polymorphisms previously reported in the human dbSNP database of the National Center for Biotechnology Information and the Ensembl database were excluded from the analysis.

### Statistical analysis

The relationship between A20 mutations with other clinicopathologic characteristics was analyzed with the χ^2^ test. Overall survival (OS) and progression-free survival (PFS) were measured from the day of diagnosis of ABC-DLBCLs and from the start day of each chemotherapeutic regimen, respectively, and these parameters were analyzed by Kaplan-Meier estimates and log-rank tests. *P* value < 0.05 were considered statistically significant. All statistical calculations were performed with SPSS 20.0 software (SPSS Inc., Chicago, IL, USA) and MedCalc software (version 9.2.0.0; Broekstraat, Mariakerke, Belgium).

## Results

### Clinical data

The clinical and pathological features of the 68 ABC-DLBCL patients are listed in Table 1 according to the presence (20 patients, 29.4%) or absence (48 patients, 70.6%) of the A20 mutation. A comprehensive correlation analysis showed that the A20 mutation was not significantly associated with clinicopathologic parameters. Most patients (55 patients, 80.9%) received anthracycline-based chemotherapy without rituximab as first-line therapy [e.g., cyclophosphamide, doxorubicin (or epirubicin) vincristine, and prednisone; CHOP]. The remaining 13 patients received rituximab-CHOP as first-line treatment. The complete remission rates were 35% and 45.8% in the A20 mutation and non-mutation groups, respectively.

### A20 gene mutations in ABC-DLBCL patients

The ABC-DLBLC clinical samples of 20 patients contained A20 gene mutations, and these mutations were characterized. Deletions were observed in 10 patients, and the most frequent mutation loci were in the ovarian tumor domain in exon 3 and the zinc finger domain in exons 7–9 (Table 2). Point mutations were found in 10 patients, and these mutations included 2 missense mutations, 1 nonsense mutation (stop codon), 1 insertion mutation (frameshift), and 3 complicated mutations (2 mutations in 1 sample). Deletion and point mutations were predicted to produce abnormal proteins that lacked domains critical for function. We also found 5 synonymous mutations, including 2 coexisting mutations with other missense mutations.

**Table 2:** Summary of A20 mutations in patients with activated B-cell-like diffuse large B-cell lymphoma.

Sequencing case number	Nucleotide position (according to NM_001270507.1)	Amino acid variant (according to NP_001257436.1)	A20 mutation type
6	G422A (exon 3) A2040G (exon 8)	R141H 680E (synonymous)	homozygous
7	Δ36bp (2544–2579) (exon 9)	truncated protein	homozygous
12	G1956A (exon 8)	Q (synonymous)	homozygous
13	Δ7bp (788–794) (exon 3)	truncated protein	homozygous
16	Δ2bp (803–804) (exon 3)	truncated protein	homozygous
18	C1725T (exon 7)	S (synonymous)	homozygous
27	Δ27bp (709–735) (exon 3)	truncated protein	homozygous
32	Δ12bp (661–672) (exon 3)	truncated protein	homozygous
34	Δ11bp (681–690) (exon 3)	truncated protein	homozygous
38	Δ3bp(2452–2454) (exon 9)	truncated protein	homozygous
41	Δ7bp(454–460) (exon 2)	truncated protein	homozygous
48	G805A (exon 5)	E269K	heterozygous
49	C326A (exon 3) G1451A (exon 7)	S109Y E484K	heterozygous
50	G1062A (exon 7)	K (synonymous)	heterozygous
52	A713G (exon 5)	W→stop codon (nonsense)	heterozygous
61	A922G (exon 6) C2050T (exon 7)	M308V 684H (synonymous)	heterozygous
65	ΔA(2202–2202), insertion (exon 9)	frameshift,734M (I)	homozygous
66	Δ5bp (2481–2485) (exon 9)	truncated protein	homozygous
67	Δ20bp (2516–2535) (exon 9)	truncated protein	homozygous
70	A829T (exon 6)	N277Y	heterozygous

### A20 mutation is not associated with poor prognosis in ABC-DLBCL patients

**Fig 1:**
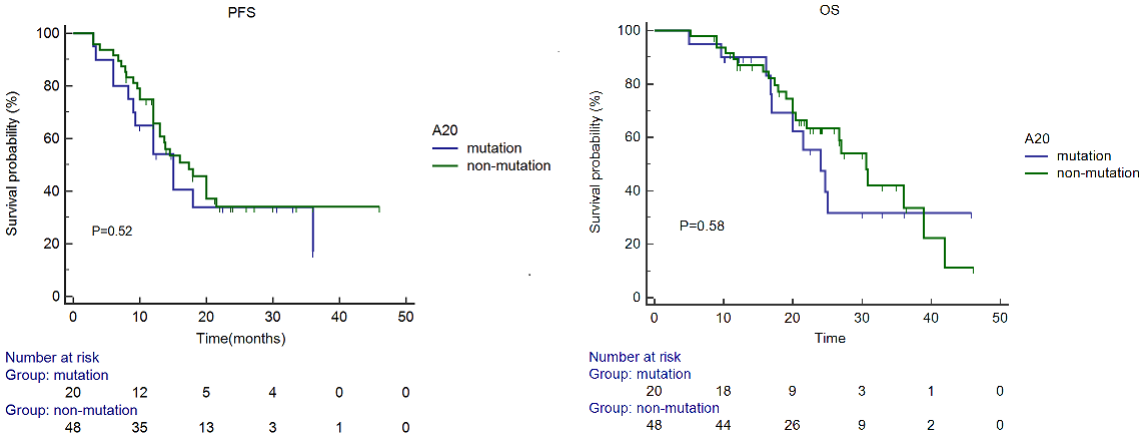
Progression-free survival (PFS) and overall survival (OS) of patients with activated B-cell-like diffuse large B-cell lymphoma with or without A20 mutations.

The median OS was 24.0 and 30.6 months in ABC-DLBCL patients with and without A20 mutations, respectively (P = 0.58) (Fig 1). The median PFS was 15 and 17.4 months in ABC-DLBCL patients with and without A20 mutations, respectively (P = 0.52). The A20 mutation had no significant impact on either OS or PFS.

## Discussion

Genome-wide expression profiling revealed the existence of different cell-of-origin classification schemes for GCB-DLBCL and ABC-DLBCL [11, 12]. Various studies have shown that this classification is an important independent prognostic factor for patients treated with CHOP or CHOP-like chemotherapy [10, 13, 14]. Distinct molecular subtypes of diffuse large-B-cell lymphoma, with CHOP-like chemotherapy, the 5-year overall survival rates of patients with ABC-DLBCL was far poor than those with GCB-DLBCL, and numerous DLBCL patients will ultimately relapse, especially those with ABC-DLBCL [11,15].

A key feature of ABC-DLBCL is activation of the NF-κB signaling pathway as evidenced by the preferential expression of NF-κB target genes in ABC-DLBCL cells and the dependence on NF-κB activity for cells proliferation and survival [1, 7]. Constitutive NF-κB activation in ABC-DLBCL is a primary pathogenetic event. Analysis of the coding sequences of genes involved in NF-κB signaling has identified mutations in genes encoding NF-κB activators, such as caspase recruitment domain-containing protein 11 [16], tumor necrosis factor receptor superfamily member 11A/receptor activator of NF-κB [17], TNF receptor-associated factor (TRAF) 2 [18,19], TRAF5 [20], AP3K/transforming growth factor beta-activated kinase 1 (TAK1) [7, 21], and the TNFAIP3/A20 NF-κB inhibitor [5,22,23]. The most commonly mutated gene was the one encoding A20, a dual function ubiquitin-modifying enzyme required for termination of NF-κB responses in the classical NF-κB pathway. This member of the ovarian tumor domain family of deubiquitinating enzymes abrogates NF-κB signaling by inactivating several proteins required for NF-κB activity, such as TAK1, TRAF6, NF-κB essential modulator, and receptor interacting protein kinase 1 [18–20]. Above investigations comfirmed that ABC-DLBCL is a molecularly heterogeneous entity and associated with multiple genes mutation. Among them, the most commonly mutated gene is A20, but the prognostic significance of A20 mutation is unclear. In this study, we investigated whether A20 mutation status could predict survival for ABC-DLBCL patients (identify two different prognostic subgroups of ABC-DLBCL).

The A20 gene is located in a chromosomal region frequently deleted in aggressive B-cell lymphomas and has been previously suggested to contain a tumor suppressor gene [24, 25]. Our study showed that the A20 mutation is a frequent event in ABC-DLBCL as follows: 23 mutations were identified in 20 of 68 ABC-DLBCL patients (Table 2). Similar to previous findings [8, 9, 26–28], most of the somatic mutations in the ABC-DLBCL samples in our study were deletion, nonsense, missense, or insertion mutations, which are all predicted to produce abnormal proteins with impaired function. Among the 20 somatic mutations, the most frequent mutation loci were located in known functional domains (ovarian tumor and zinc finger). We also identified 5 synonymous mutations, and 2 of these mutations were concurrent with other missense mutations. Recent studies have shown that both synonymous and non-synonymous mutations affect mRNA stability, mRNA processing, and mRNA maturation, which consequently affect allelic mRNA expression, protein abundance, and function [29–31]. Synonymous mutations have also been hypothesized to introduce a rare codon that affects the timing of co-translational folding, thereby altering the structure of substrate and inhibitor interaction sites [32]. Whether synonymous mutations also impair A20 protein function has yet to be tested. Previous studies confirmed that ABC-DLBCL was associated with A20 mutation [8, 9, 26–28], Dong et al. [33] showed that the A20 somatic mutation significantly correlates with poor OS and event-free survival in gastrointestinal DLBCL. Importantly, however, the study by Dong et al. included GCB-DLBCL patients, the survival difference between patients with or without A20 mutation could theoretically be related to the differences between ABC and GCB, since most patients with A20 mutation was in ABC-DLBCL subgroup. In another investigation about the prognostic role of A20 mutation in DLBCL, Paik et al. [34] make a conclusion that A20 deletion was not a poor prognostic factor for DLBCL,but this conclusion need to be carefully interpreted, because in their study, A20 deletion rate was high (23.1%) in GCB-DLBCL and similar to ABC-DLBCL, which was contradictory to previously researches.

. The present study, which included only ABC-DLBCL patients, showed that the A20 mutation was not significantly associated with PFS or OS. Mutations in other genes could theoretically activate NF-κB signaling in ABC-DLBCL. Therefore, the A20 mutation may only partly account for NF-κB activation in this disease, Mutations in genes other than A20 may be present in the non-mutation group. It is also possible that the relatively small number of patients in the present study precluded accurate survival analysis.

Some limitations for this study include, first, the analyses based on retrospective cohort studies other than prospective randomized trials; second, most patients in this analysis received anthracycline-based chemotherapy without Rituximab as first-line therapy, we did not perform subgroup analysis for patients received CHOP or R-CHOP according to A20 mutation status because the number of included cases was small, and the effect of Rituximab on our study is unclear; third, small sample sizes may be difficult to detect difference between A20 mutation group and non-A20 mutation group.

In conclusion, we showed that the A20 mutation is a frequent event in ABC-DLBCL. However, there was no significant difference in PFS and OS in patients with or without A20 mutations. Further study is required to completely exclude A20 somatic mutation as a prognostic marker in the ABC subtype of DLBCL.

## Supporting Information

S1 Fig PCR amplicons for each primer sets on gel. (DOC)

S2 Fig Examples of A20 mutation detected by PCR and DNA sequencing. (DOC)

S3 Fig Schematic diagram of A20 gene and protein to represent mutation according to the corresponding protein functional domains. (DOC)

S4 Fig Immunohistochemical result of one representative case of ABC DLBCL (LSAP immunostaining; 400×). (PDF)

S1 Table Primers and PCR conditions used for amplification of the A20 coding exons. (DOC)
