# Supplementary material for: A20 Mutation Is Not a Prognostic Marker for Activated B-Cell-Like Diffuse Large B-Cell Lymphoma
Source: PLoS One. 2015 Dec 30;10(12):e0145037. doi: 10.1371/journal.pone.0145037 (PMC4696786; doi:10.1371/journal.pone.0145037)

**S2 Fig. PCR amplicons for each primer sets on gel (Figure A, Figure B, Figure C and Figure D).**

**Figure A, PCR amplicons** **on gel (Exon2A to Exon6A)**


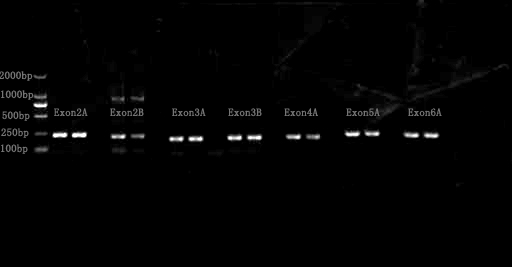


**Figure B, PCR amplicons** **on gel (Exon7A to Exon7F)**


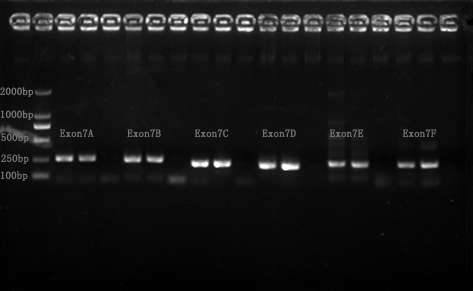


**Figure C, PCR amplicons** **on gel (Exon7G to Exon9A)**


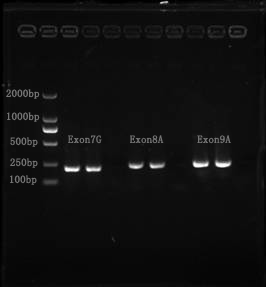


**Figure D, PCR amplicons** **on gel (Exon9B to Exon9C)**


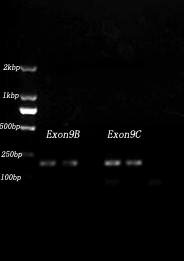

Supplement: S1 Fig — (DOC) [file pone.0145037.s001.doc]
