# Supplementary material for: A20 Mutation Is Not a Prognostic Marker for Activated B-Cell-Like Diffuse Large B-Cell Lymphoma
Source: PLoS One. 2015 Dec 30;10(12):e0145037. doi: 10.1371/journal.pone.0145037 (PMC4696786; doi:10.1371/journal.pone.0145037)

**S3 Fig. Examples of A20 mutation detected by PCR and DNA sequencing.**

**Case52, exon5(A713G, heterozygous）(W123→stop codon)**


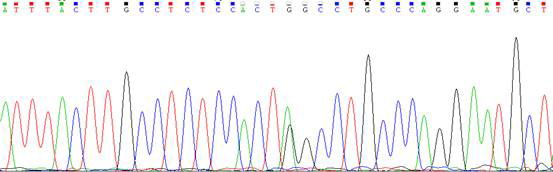


**Case6, exon3（G422A, homozygous）(R141H)**


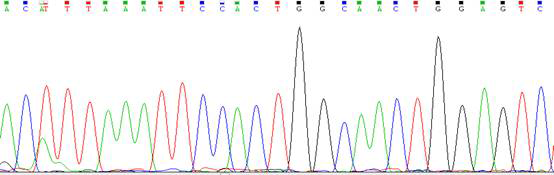


**Case16, Normal DNA, exon3**


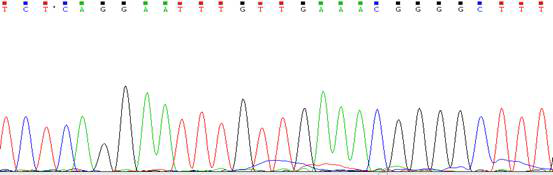


**Case16, exon3, △GT (803-804)**


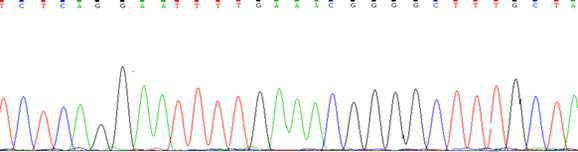

Supplement: S2 Fig — (DOC) [file pone.0145037.s002.doc]
