# Supplementary material for: A20 Mutation Is Not a Prognostic Marker for Activated B-Cell-Like Diffuse Large B-Cell Lymphoma
Source: PLoS One. 2015 Dec 30;10(12):e0145037. doi: 10.1371/journal.pone.0145037 (PMC4696786; doi:10.1371/journal.pone.0145037)
Supplement: S3 Fig — (DOC) [file pone.0145037.s003.doc]

**S4 Fig. Schematic diagram of A20 gene and protein to represent mutation according to the corresponding protein functional domains**


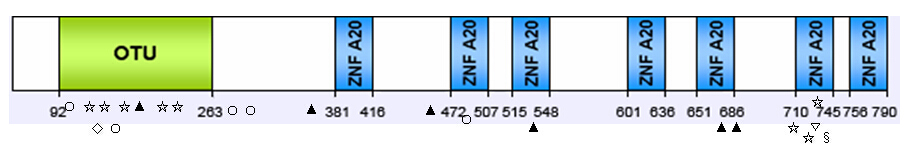


▲synonymous mutations; ▽deletion mutations; ☆ truncated protein; ◇ nonsense mutation; ○ missense mutation; §insertions mutations.
