# Supplementary material for: A20 Mutation Is Not a Prognostic Marker for Activated B-Cell-Like Diffuse Large B-Cell Lymphoma
Source: PLoS One. 2015 Dec 30;10(12):e0145037. doi: 10.1371/journal.pone.0145037 (PMC4696786; doi:10.1371/journal.pone.0145037)
Supplement: S4 Fig — (PDF) [file pone.0145037.s004.pdf]

Example of immunohistochemical staining (Case 8)

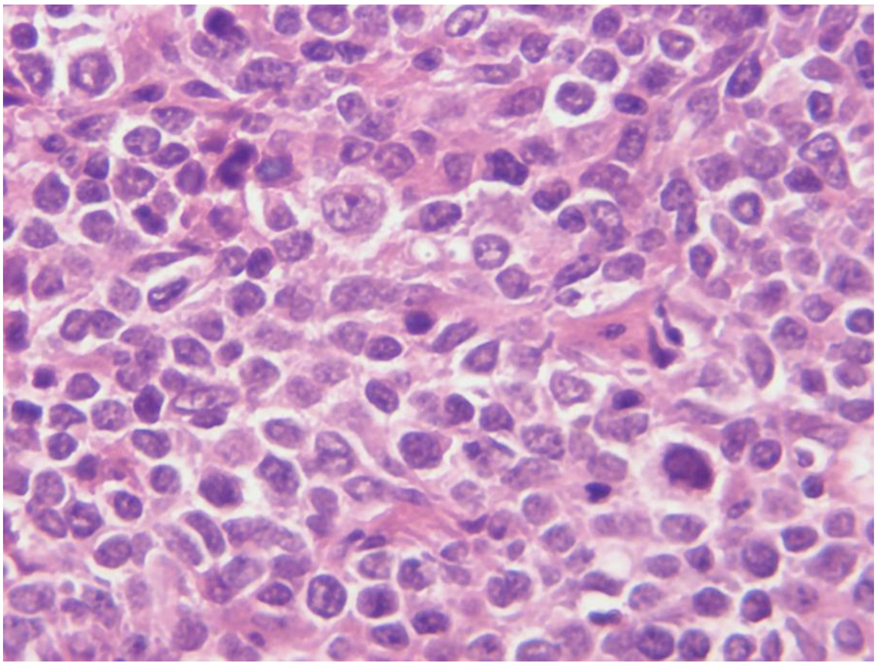

HE400X

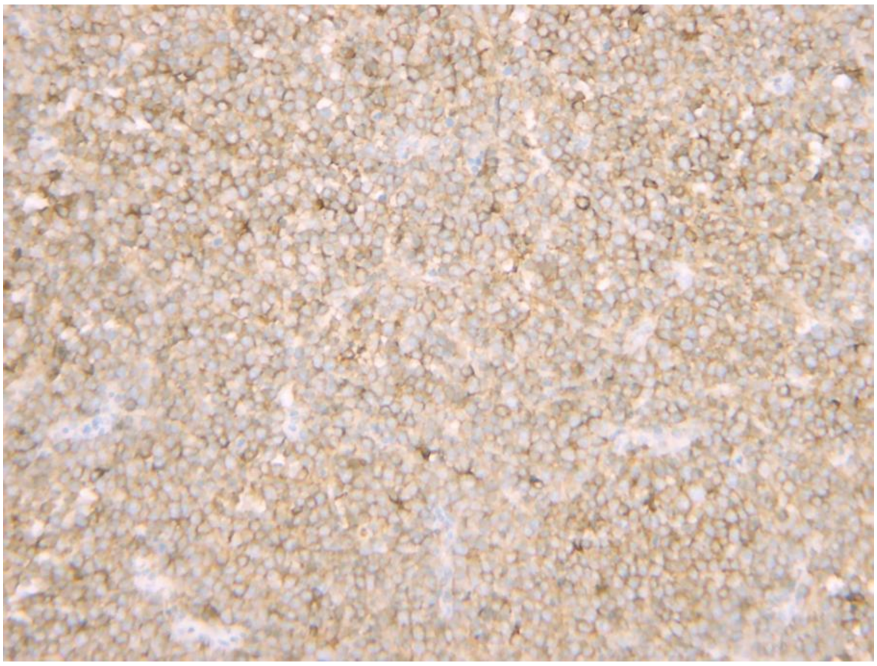

CD20(positive ）

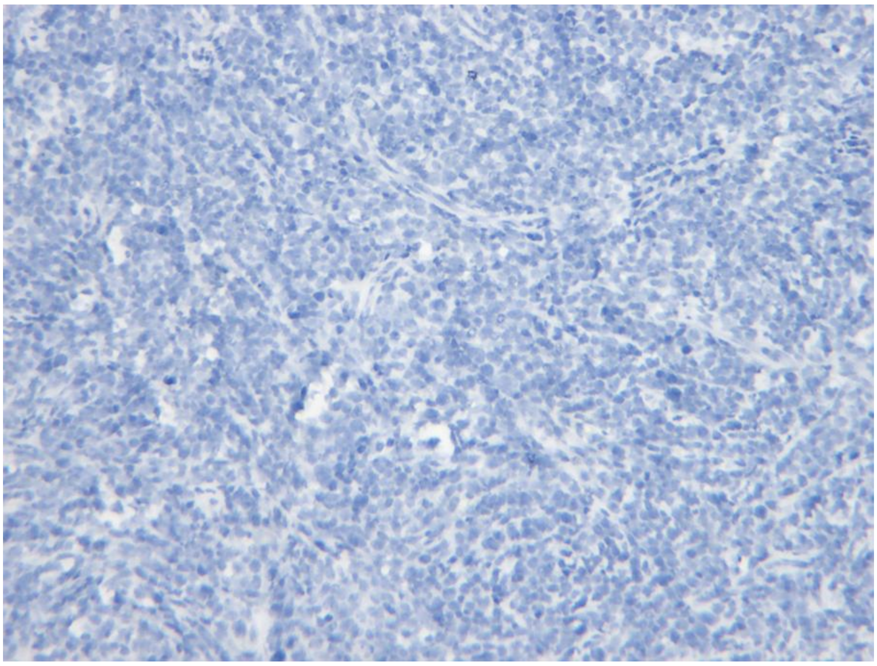

CD10(negative)

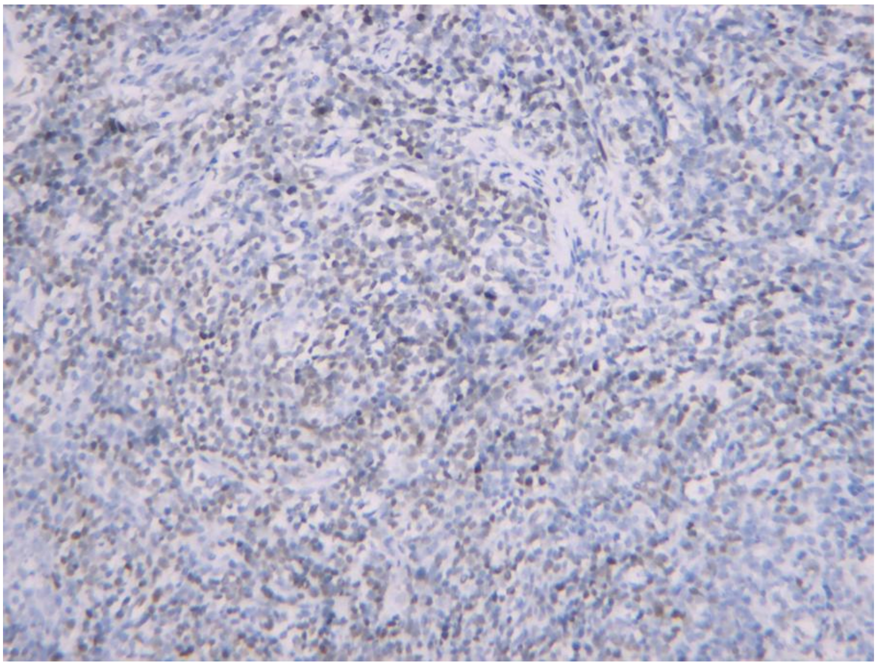

BCL-6(positive)

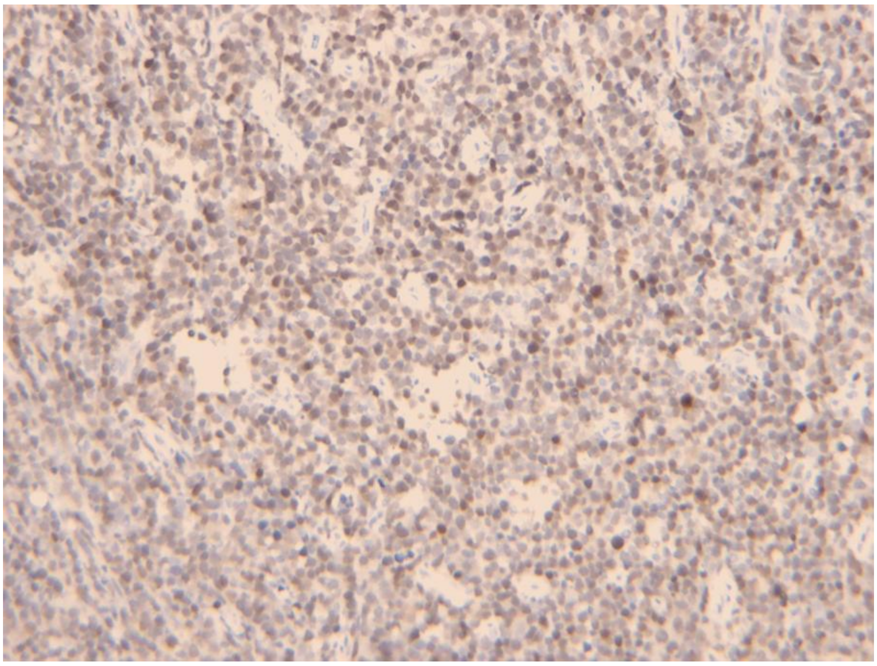

MUM-1(positive ）
