# Supplementary material for: A20 Mutation Is Not a Prognostic Marker for Activated B-Cell-Like Diffuse Large B-Cell Lymphoma
Source: PLoS One. 2015 Dec 30;10(12):e0145037. doi: 10.1371/journal.pone.0145037 (PMC4696786; doi:10.1371/journal.pone.0145037)
Supplement: S1 Table — (DOC) [file pone.0145037.s005.doc]

S1 Table. Primers and PCR conditions used for amplification of the A20 coding exons

| Gene | Exon | Primer name | Sequence（5’-3’） | Amplicon size（bp） | PCR condition |
| --- | --- | --- | --- | --- | --- |
| A20 | Exon2 | A20-E2A-F | TCCTTTTCAGGTGTTGGAGAG | 232 | PCR was carried out in 25μl reaction mixture with 10ng template DNA in an ABI 3730 analyser (Life technology, Japan).  The PCR conditions were 95°C for 3min, followed by 10 touchdown cycles consisting of 95°C×10 sec, 62°C×20 sec,72°C×25 sec, then 35 cycles of 95°C×10 sec, 57°C×20 sec and 72°C×25 sec. |
|  |  | A20-E2B-R | GATGAGGGCTTTGTGGATG |  |  |
|  |  | A20-E2C-F | ACCATGCACCGATACACAC | 192 |  |
|  |  | A20-E2C-R | CACAACAGAACAAGTCTTACCG |  |  |
|  | Exon3 | A20-E3A-F | CAGTAGGGCTGGTTTATTCTG | 206 |  |
|  |  | A20-E3A-R | GTTGCGTGTGTCTGTTTCC |  |  |
|  |  | A20-E3B-F | TGCATGCCACTTCTCAGTAC | 210 |  |
|  |  | A20-E3B-R | GAGCTCTGTTAGTAGATAATTAGGG |  |  |
|  | Exon4 | A20-E4A-F | CCCTTAGAACTGGAATGATGAATG | 247 |  |
|  |  | A20-E4A-R | TACCTGAACTTTGGCAGTGTG |  |  |
|  | Exon5 | A20-E5A-F | CCTCATTTTCCTTTCTCTTTCTTTG | 236 |  |
|  |  | A20-E5A-R | CTAGTTATAGATGTGAATTAATGCAG |  |  |
|  | Exon6 | A20-E6A-F | TGGCCTTGTTTAGTAGAATACTG | 262 |  |
|  |  | A20-E6A-R | CAGGAGAGAGCTGAACATAAAC |  |  |
|  | Exon7 | A20-E7A-F | CTTGCCATAATCCACATTCTAAAAC | 247 |  |
|  |  | A20-E7A-R | GAAGGTTCCATGGGATTCTG |  |  |
|  |  | A20-E7B-F | CAATCTGGTAGATGATTACTTTGAAC | 215 |  |
|  |  | A20-E7B-R | GCCTCTCTGAGCACTCATG |  |  |
|  |  | A20-E7C-F | CCAGCTTTCTCTCATGGATGTA | 205 |  |
|  |  | A20-E7C-R | CAAGGGCTCATAGGCTTCTC |  |  |
|  |  | A20-E7D-F | CAAACTCCCAAAGCTGAACTC | 230 |  |
|  |  | A20-E7D-R | CGTTGTGCTGCACATTCAG |  |  |
|  |  | A20-E7E-F | ACCCAGCCCTTTTCTGTTC | 229 |  |
|  |  | A20-E7E-R | GAAGCAAGTACTGCAGATCC |  |  |
|  |  | A20-E7F-F | ACGGATTTTGTGAACGTTGC | 272 |  |
|  |  | A20-E7F-R | AGCTCTGTGGCAAGAATGC |  |  |
|  |  | A20-E7G-F | CATTCTTGCCACAGAGCTG | 208 |  |
|  |  | A20-E7G-R | CTGTGTTAGGAAGTCAACCAC |  |  |
|  | Exon8 | A20-E8A-F | CCCTATGTGGTACTAACTAGCATC | 241 |  |
|  |  | A20-E8A-R | AAAGCTCCTCCAAGTGTCTC |  |  |
|  | Exon9 | A20-E9A-F | CTCTTTGTAGACTCCACACTC | 233 |  |
|  |  | A20-E9A-R | TGCTGACACTCCATGCAG |  |  |
|  |  | A20-E9B-F | CTCGAACCACACAAAGCAC | 212 |  |
|  |  | A20-E9B-R | CTTGAACTGAAAGCATTCGTTG |  |  |
|  |  | A20-E9C-F | CAACGAATGCTTTCAGTTCAAG | 210 |  |
|  |  | A20-E9C-R | GCACCACGAAGAGCTTATC |  |  |
